# Anti-Inflammatory Activity of Isomaltodextrin in a C57BL/6NCrl Mouse Model with Lipopolysaccharide-Induced Low-Grade Chronic Inflammation

**DOI:** 10.3390/nu11112791

**Published:** 2019-11-15

**Authors:** Melissa Hann, Yuhan Zeng, Lingzi Zong, Takeo Sakurai, Yoshifumi Taniguchi, Ryodai Takagaki, Hikaru Watanabe, Hitoshi Mitsuzumi, Yoshinori Mine

**Affiliations:** 1 Department of Food Science, University of Guelph, Guelph, ON N1G2W1, Canada; hannm@uoguelph.ca (M.H.); yzeng05@uoguelph.ca (Y.Z.); lzong@uoguelph.ca (L.Z.); 2 R&D Center, Hayashibara CO., LTD., 675-1 Fujisaki, Naka-ku, Okayama 702-8006, Japan; takeo.sakurai@hb.nagase.co.jp (T.S.); yoshifumi.taniguchi@hb.nagase.co.jp (Y.T.); ryodai.takagaki@hb.nagase.co.jp (R.T.); hikaru.watanabe@hb.nagase.co.jp (H.W.); hitoshi.mitsuzumi@hb.nagase.co.jp (H.M.)

**Keywords:** isomaltodextrin, low-grade chronic inflammation, lipopolysaccharide, pro-inflammatory mediators, adipocyte, PPAR-γ, mucin, tight junction proteins, microbiota, short chain fatty acids

## Abstract

**The** purpose of this study was to identify the anti-inflammatory activity and mechanism of isomaltodextrin (IMD) in a C57BL/6NCrl mouse model with lipopolysaccharide (LPS)-induced systemic low-grade chronic inflammation and the effect on inflammation-induced potential risk of metabolic disorders. Pre-treatment of IMD decreased the production of pro-inflammatory mediators, TNF-α and MCP-1, and stimulated the production of the anti-inflammatory mediator, adiponectin by increasing the protein expression of peroxisome proliferator-activated receptor gamma (PPAR-γ) in the white adipose tissues. IMD administration reduced plasma concentrations of endotoxin, decreased macrophage infiltration into adipocytes, and increased expression of mucin 2, mucin 4, and the tight junction protein claudin 4. These results suggest that IMD administration exerted an anti-inflammatory effect on mice with LPS-induced inflammation, potentially by decreasing circulating endotoxin, suppressing pro-inflammatory mediators and macrophage infiltration, or by improving mucus or tight junction integrity. IMD exerted protein expression of insulin receptor subset-1 (IRS-1). IMD alleviated the disturbance of gut microflora in LPS-treated mice, as the number of *B. bifidum*, *L. casei,* and *B. fragilis* increased, and *E. coli* and *C. difficile* decreased, when compared to LPS-treated mice. The analysis of short chain fatty acids (SCFAs) further supported that the concentrations of acetic and butyric acids were positively correlated with IMD, as well as the number of beneficial bacteria. This study provides evidence that IMD possesses anti-inflammatory properties and exerts beneficial functions to prevent systemic low-grade chronic inflammation and reduces the risk of developing insulin resistance and associated metabolic diseases.

## 1. Introduction

The gastrointestinal (GI) tract provides a selectively permeable barrier that performs two major functions. First, the breakdown and absorption of nutrients from food occur throughout the length of the GI tract [1]. Second, it prevents the movement of harmful material, including antigens, pathogens, and toxins, from the lumen into the intestinal tissue [2]. The epithelium must be selectively permeable to allow the passage of various nutrients, ions, and water, while preventing the passage of other luminal content. Solute movement typically incorporates transcellular transport using various selective transporters, but paracellular transport of ions and solutes may also occur [1,3]. The paracellular pathway between cells is typically made highly selective by the presence of a tight junction (TJ) protein complex, which connects two adjacent epithelial cells [4]. Intestinal permeability may be increased due to decreased expression or redistribution of TJ proteins, or a reduction in the number of goblet cells, leading to a thinner mucus layer [5,6]. A disrupted mucosal barrier can then allow pathogens to pass through the paracellular space and enter intestinal tissue.

Under normal circumstances, the inflammatory response is beneficial because it provides a defense against invading pathogens and repairs tissue damage, however, it is essential that the inflammatory response is resolved in a timely manner [7]. Low grade chronic inflammation is defined as a persistent state of increased levels of pro-inflammatory cytokines. These cytokines are present at a lower level than found in inflammation, with approximately a 2- to 3-fold elevation of pro-inflammatory mediators systemically [8]. Low-grade chronic inflammation is typically asymptomatic, leaving the condition undiagnosed. One potential cause is an increase in intestinal permeability that may allow increased levels of lipopolysaccharide (LPS) or other pathogen-associated molecular pattern (PAMPs) and induce inflammation throughout the body [6]. 

Low-grade chronic inflammation may contribute to various metabolic disorders, such as obesity and insulin resistance [9]. In many cases, obesity is strongly associated with low-grade chronic inflammation of the white adipose tissue (WAT) [10]. WAT contains adipocytes and macrophages, which are both capable of releasing pro-inflammatory or anti-inflammatory cytokines. An improper balance of pro-inflammatory and anti-inflammatory cytokines is commonly found in obese adipose tissue and may contribute to the development of other diseases [11]. Serum levels of adiponectin are decreased with an increased fat mass, as well as in patients with increased inflammatory states, such as those with cardiovascular disease [12]. Adiponectin has also been shown to inhibit the expression of pro-inflammatory mediators, such as tumor necrosis factor-α (TNF-α), interleukin-6 (IL-6), monocyte chemoattractant protein-1 (MCP-1), and IL-1β, as well as promoting the expression of the anti-inflammatory cytokine IL-10 [13]. Adiponectin signaling inhibits the nuclear factor kappa-light-chain enhancer of activated B cells (NF-κB) and enhances peroxisome proliferator-activated receptor gamma 2 (PPAR-γ2), which can suppress the transactivation capability of NF-κB [13].

Insulin resistance is defined as a smaller than normal response to a constant amount of insulin [14]. The insulin receptor substrate (IRS) proteins are essential for continuing the signal cascade that culminates in increased glucose transport. The primary method of insulin-mediated glucose transport is through the translocation of glucose transporters, especially glucose transporter type 4 (GLUT4), from intracellular vesicles to the cell membrane [15,16]. IRS-1 plays an important role in insulin-stimulated GLUT4 translocation, as this translocation is greatly reduced in the presence of the same levels of insulin relative to a control if IRS-1 is not produced [16,17]. The pro-inflammatory cytokines TNF-α and IL-6 have previously been demonstrated to inhibit this insulin signaling pathway [10]. Insulin resistance is also associated with diseases such as type 2 diabetes, non-alcoholic fatty liver disease, and coronary heart disease [18,19].

Another important aspect for gut health is the composition of the gut microbiome. An estimated 10^14^ bacteria reside in the human colon, which contains more commensal bacteria than any other organ [20]. There are approximately 7000 strains and 400 species of bacteria present in the colon, with many of the predominant members being obligate anaerobes, such as *Bacteroides*, *Bifidobacterium*, *Clostridium*, *Enterococcus*, *Eubacterium*, *Fusobacterium*, *Peptococcus*, *Peptostreptococcus*, *Lactobacillus*, and *Ruminococcus* [21,22]. Gut microbiota can have an important impact on gut health in various ways. For example, bacteria can ferment non-digestible saccharides into short chain fatty acids (SCFAs), which can encourage the proliferation of epithelial cells, mucosal blood flow, and colonic motility. Butyrate is the major energy source for colonic epithelial cells. Furthermore, there is evidence demonstrating that SCFAs can reduce the permeability of cecal mucosa [23]. Commensal bacteria may also prevent the colonization of pathogenic bacteria by competing for nutrients or adhesion sites, as well as lowering the pH, thereby inhibiting growth [24]. In order to maintain a healthy gut, it is important to have a greater quantity of beneficial bacteria, such as *Bifidobacteria* and *Lactobacilli*, relative to potentially harmful or pathogenic bacteria [25]. 

Bioactive food compounds, such as polyunsaturated fatty acids (PUFAs), vitamins, polyphenols, probiotics, peptides, amino acids, and prebiotics all demonstrate anti-inflammatory activity. In general, these mechanisms may include the inhibition of pro-inflammatory mediator expression, the promotion of anti-inflammatory mediator expression, as well as interfering with signaling pathways, especially the NF-κB pathway, and activating PPAR-γ. Prebiotics are defined as “a substrate that is selectively utilized by host microorganisms conferring a health benefit” [26]. Additionally, dietary prebiotics must demonstrate resistance to digestion by gastric acidity and mammalian enzymes, as well as absorption in the GI tract, the ability to be fermented by intestinal microbiota, and the selective stimulation of growth or activity of beneficial bacteria in the intestine. The most commonly used prebiotics are fructans, including inulin and oligofructose, and galactans, such as galactooligosaccharides (GOSs) [25,26]. These compounds are selectively fermented by bifidobacteria because they are easily digested by β-fructanosidase and β-galactosidase, respectively, and these enzymes are prevalent in bifidobacteria [26]. Inulin-type fructans are one of the most common types of fructans used as prebiotics

Isomaltodextrin (IMD) is a highly branched, water soluble α-glucan that has demonstrated resistance to digestion by mammalian enzymes [27]. It consists of glucose residues with many α-1,4-, α-1,6-, and α-1,3,6-linkages (supplemental Figure S1). IMD is enzymatically produced from maltodextrin by the enzymes α-glucosidase and α-amylase isolated from the bacterial strain *Paenibacillus* sp. PP710 [28]. Sadakiyo et al. have demonstrated that IMD can benefit human health, through its actions as a soluble dietary fiber, by suppressing glucose absorption in the small intestine and attenuating blood glucose elevation following a meal [29]. Furthermore, IMD may have the ability to act as a prebiotic, due to its resistance to digestion by β-amylase [27]. In a study by Nishimura et al., the colonic fermentation of IMD was investigated in rats [30]. It was determined that IMD increases hydrogen excretion in breath and flatus, in a dose-dependent manner, to a similar level as fructooligosaccharides, a proven prebiotic. Hydrogen excretion can be an indicator of colonic hydrogen production, indicating that IMD is fermented in the colon. Furthermore, Nishimura et al. also investigated the number of *Bifidobacteria* in the cecum and found a dose-dependent increase, suggesting that IMD may be a bifidogenic α-glucan [30]. The efficacy of treatment with IMD for intestinal inflammation was also investigated in a mouse model of colitis [31]. Treatment with IMD did not improve typical colitis symptoms, however, IMD treatment was associated with a significant reduction in the expression of pro-inflammatory mediators TNF-α and IL-8, as well as TLR4. This indicates that IMD may have anti-inflammatory activity [31]. However, the effect of IMD on gut barrier functions and prevention of potential risk of metabolic disorder marker (especially targeting obesity and insulin resistance) has not been elucidated.

For that, this study aimed to evaluate the anti-inflammatory properties of IMD and assess its effect on metabolic disorders induced by inflammation namely obesity and insulin resistance using a C57BL/6NCrl mouse model with LPS-induced systemic low-grade chronic inflammation.

## 2. Materials and Methods

### 2.1. Animal study

This animal study was approved by the University of Guelph Animal Care Committee in accordance with the Canadian Council on Animal Care Guide to the Care and Use of Experimental Animals. The Animal Utilization Protocol (AUP) number for the animal study is AUP3502. Fifty-two female C57BL/6NCrl mice (15–16 weeks, 15–22 g; Charles River Laboratories, Montreal, QC) were used in this animal study. The mice were housed four per cage. The mice were randomly divided into five intervention groups, with the negative (NC) and positive (PC) control groups each having 8 mice, and 12 mice in each of the low dose (1.0%: TL), medium dose (2.5%: TM), and high dose (5.0%: TH) treatment groups. IMD (Fibryxa from Hayashibara, Ltd (Okayama, Japan)) and LPS isolated from *Escherichia coli* strain O111:B4 (Sigma, Oakville, ON) were introduced through the autoclaved drinking water. Experimental design, treatments’ assignment, and duration are presented in Table 1. 

### 2.2. Glucose Tolerance Test

Two mice from each cage, mouse one and two, were selected to undergo the glucose tolerance test, for a total of 30 mice (4–6 mice in each group). Six hours before the test was performed, mice were fasted by removing food from the cages. Initial blood glucose levels were measured by cutting the tip off the mouse’s tail and then collecting the drop of blood on a test strip for the Onetouch Ultra2 glucose meter (ONETOUCH Ultra, Burnaby, Canada). Immediately following the initial reading, the mice underwent a glucose gavage of 150 μL of 2 g glucose (kg/body weight) (Sigma-Aldrich, Oakville, ON, Canada) in autoclaved water. Blood glucose levels were taken for each mouse at 15, 30, 60, and 120 minutes after the glucose gavage. Immediately following the reading at 120 minutes, mice were euthanized by CO_2_ asphyxiation. Results were used to determine glucose tolerance. 

### 2.3. Endotoxin and D-mannitol Assays

Plasma endotoxin concentrations were measured using the Pierce LAL Chromogenic Endotoxin Quantitation Kit (Thermo Scientific, Whitby, ON, Canada) according to the manufacturer’s instructions. The plate was kept at 37 °C in a heating block throughout the reaction. Standards were diluted according to instructions to give a range of 0.1 EU/mL-1 EU/mL. Standards and samples were added directly to a preheated plate and incubated for 5 minutes before 50 μL/well of *Limulus* Amebocyte Lysate (LAL) was added. The plate was incubated for 10 minutes, then 100 μL of chromogenic substrate was added. The reaction was allowed to continue at 37 °C until sufficient color developed, at which point 50 μL of acetic acid was added to each well and absorbance was measured at 410 nm by a microplate reader (iMark model 550, Bio-Rad Laboratory, Mississauga, ON).

The intestinal permeability of two mice from each cage was investigated using a D-mannitol test. Mice were fasted 6 hours before the test. For the test, the mice each received an oral gavage of 150 μL of 0.6 g/ kg body weight D-mannitol (Sigma) in autoclaved water. Two hours after the gavage, mice were euthanized by CO_2_ asphyxiation and blood was immediately collected from the heart and stored in EDTA coated tubes before separating the plasma by centrifuge (2500× *g*, 4 °C for 20 minutes). Plasma concentrations of D-mannitol were determined by colorimetric assay kit (Abcam Inc. Toronto, Canada) and used as an index of intestinal permeability.

### 2.4. Plasma Collection

Immediately following euthanasia, blood was collected by cardiac puncture and stored temporarily in EDTA-coated tubes on ice. The tubes were centrifuged (2500× *g*, 4 °C for 20 minutes), then plasma was aspirated into a 1.5 mL screw-cap microtube (Sarstedt, Nümbrecht, Germany). Plasma samples of the two mice from the same cage that underwent the same test, glucose tolerance or intestinal permeability, were pooled together, due to the limited volume of plasma available and were flash-frozen in liquid nitrogen before being stored at –80 °C for further analysis.

### 2.5. Tissue Collection

Abdominal white adipose tissue was collected, weighed, and divided into two 1.5 mL screw-cap microtubes (Sarstedt). One tube was flash frozen in liquid nitrogen before being stored at −80 °C for Western blot analysis. The other tube contained 10% formalin, and the white adipose tissue from this tube was used for immunohistochemical analysis. The jejunum was collected and stored in RNAlater™ solution (Invitrogen, Burlington, ON, Canada) at −80 °C for future real-time polymerase chain reaction (RT-PCR) analysis. The liver, kidney, heart, leg muscle, and brown adipose tissue were also collected, weighed, flash frozen in liquid nitrogen, and stored at −80°C.

### 2.6. Enzyme-Linked Immunosorbent Assay (ELISA)

Commercially available ELISA kits were used to measure plasma concentrations of adiponectin, TNF-α, and MCP-1 (eBioscience, Affymetrix, Inc. San Diego, CA, USA), according to the manufacturer’s instructions. In brief, 96-well high binding surface microplates (Corning, Corning, NY) were coated with 100 μL per well of capture antibody diluted in coating buffer and incubated overnight at 4 °C. Following this, plates were washed four times with phosphate-buffered saline-0.05% Tween 20 (PBST) with the BioTek microplate washer (BioTek, Winooski, VT). Blocking was performed by incubating the microplate with 200 μL per well of 1X ELISA diluent at 37 °C on an orbital shaker for one hour. Standards were prepared for each ELISA assay as per manufacturer directions before washing the plates four times. The standard ranges were as follows: 15.625–1000.00 pg/mL for TNF-α and MCP-1, and 31.25–2000.00 pg/mL for adiponectin. The diluted standards or plasma samples were added in duplicate to the designated wells at a volume of 100 μL per well and incubated for two hours at 37 °C with gentle shaking. The microplates were then washed four times before incubating for one hour at 37 °C with 100 μL/well of detection buffer diluted in 1× ELISA diluent. After washing four times, 100 μL of avidin horseradish peroxidase diluted in 1× ELISA diluent was added to each well and the plate was incubated at 37 °C for 30 minutes with gentle shaking. The microplates were then washed four more times and 100 μL per well of 1× 3,3’,5,5’-tetramethylbenzidine (TMB) substrate solution was added and incubated at 37 °C. Once sufficient color had developed, the TMB reaction was terminated by adding 50 μL of 2 N sulphuric acid to each well. Finally, the absorbance was read by a microplate reader (iMark model 550) at 450 nm. Following protein extraction, white adipose tissue adiponectin concentrations were measured by an ELISA kit according to the manufacturer’s instructions, as described above. 

Plasma concentrations of insulin were also calculated using the Ultra-Sensitive Mouse Insulin ELISA Kit (Crystal Chem, Elk Grove Village, IL, USA) according to the manufacturer’s instructions. The standard range was 0.1 ng/mL−6.4 ng/mL and the kit contained microplate wells pre-coated with antibody. The samples and standard were added directly to the wells (5 μL of sample or standard in 95 μL of diluent) and incubated for 2 hours at 4 °C before washing five times. An anti-insulin enzyme conjugate was then added to each well (100 μL) and incubated for 30 minutes at room temperature. The plate was washed seven times and 100 μL/well of substrate was added and allowed to react at room temperature for 40 minutes before stop solution was added and the absorbance at 450 nm was measured.

### 2.7. Analysis of Gene Expression by RT-PCR 

#### 2.7.1. RNA Extraction and Purification

Approximately 50 mg of mouse jejunum was weighed into 1.0 mL of TRIzol® (Invitrogen) and cut into small pieces before being homogenized (Polytron PT 1200, Kinematica, AG, Luzern, Switzerland) for 1 minute. The homogenized tissue samples were centrifuged at 12,000 g for 10 minutes at 4 °C and 650 μL of the supernatant was transferred to a fresh tube. The remaining supernatant was placed in an additional tube and stored at −80 °C. Then, 130 μL of chloroform was added to 650 μL of supernatant and shaken for 30 seconds before incubating at room temperature for 3 minutes. The samples were then centrifuged at 12,000 g for 15 minutes at 4 °C and the supernatant was transferred to a fresh tube. An equal volume of 70% ethanol was added and mixed by pipetting before transferring the entire solution to a spin column.

The Aurum Total RNA Mini Kit (Bio-Rad) was used for purification of the RNA. The spin column was centrifuged for 1 minute and the filtrate discarded. Low stringency wash solution (700 μL) was added, then the column was centrifuged for 1 minute and the filtrate discarded. Following this, 80 μL of diluted DNase I was added to the column and incubated for 25 minutes at room temperature. The column was centrifuged for 1 minute and the filtrate discarded before 700 μL of high stringency wash solution was added and the spin column centrifuged for 1 minute. The filtrate was discarded and 700 μL of low stringency wash solution was added and the column was centrifuged for 1 minute, the filtrate discarded, and the column centrifuged for 1 additional minute. The spin column was placed in a fresh tube and 60 μL of elution solution heated to 70°C was added to the column. It was incubated for 1 minute at room temperature before being centrifuged for 2 minutes. A spectrophotometer (NanoDrop® ND-1000, Thermo Scientific, Wilmington, DE) was used to determine the quality and quantity of RNA by measuring absorbance at 260 nm. The A260/A280 ratio was used to verify RNA quality, with a ratio of approximately 2 deemed acceptable.

#### 2.7.2. cDNA Synthesis

Reverse transcription of mRNA to cDNA was carried out using the High-Capacity cDNA reverse transcription kit (Applied Biosystems, Foster City, CA) according to the manufacturer’s instructions. RNase inhibitors were added to the reaction and RNA was added to yield a final concentration of 100 ng/μL in the reaction mix. Thermal cycling was performed on the MyCycler Thermocylcer (Bio-Rad) under the conditions presented in Supplemental Table S1.

#### 2.7.3. Analysis of Gene Expression in Jejunum

RT-PCR was performed using the RT SYBR Green qPCR Master Mix (SA Biosciences) on a MyIQ Single Color Real-Time PCR Detection System (Bio-Rad). Thermal cycling conditions are presented in Supplemental Table S2. The University of Guelph Laboratory Services Molecular Biology Section (Guelph, ON, Canada) synthesized the primers and primer sequences are presented in Supplemental Table S3. Relative gene expression was calculated as 2^-ΔΔCT^ using glyceraldehyde-3 phosphate dehydrogenase (GAPDH), as the reference gene and results were presented as the fold expression change relative to the negative control (NC) group [32].

### 2.8. Protein Extraction

Protein extraction from white adipose tissue was performed using the Minute™ Total Protein Extraction kit for Adipose Tissue (Invent, Plymouth, MN) according to the manufacturer’s directions. Adipose tissue was thawed, and the oil was patted out with layers of Kim wipes before weighing 100 mg into a microfuge tube and adding 100 mg of extraction powder directly on top of the tissue. A 100× dilution of protease inhibitor in extraction buffer A was prepared and 50 μL of this solution was added to the adipose tissue. The tissue was ground with a pestle for two minutes before an additional 200 μL of the extraction buffer/ protease solution was added and the tissue was ground for another 30 seconds. The tissue lysates were centrifuged for one minute at 380 g and the supernatant was transferred to a filter cartridge. The lysate in filter cartridge was incubated at −20 °C for 20 minutes with the cap open before being centrifuged at 380 g for two minutes. The protein concentration was measured using the DC Protein Assay (Bio-Rad) and samples were diluted to 4 mg/mL. An equal volume of Laemmli 2× sample buffer was added and each sample was centrifuged at 2375 g for two minutes before heating at 95 °C for five minutes. Samples were finally centrifuged at 16,000 g for one minute and stored at −80 °C for Western blot.

### 2.9. Western Blot

Extracted protein from the white adipose tissue samples were added to sodium dodecyl sulfate-polyacrylamide gel electrophoresis (SDS-PAGE) gels consisting of 10% resolving gel and 4% stacking gel. For the detection of PPAR-γ and TLR-4, 20 μg of protein was added to each lane, and for the detection of IRS-1, 30 μg of protein was added. Samples were separated at 50 V for 30 minutes followed by 100 V for two hours. Immediately following this, samples were transferred from the SDS-PAGE gel to a nitrocellulose membrane (Bio-Rad) at 100 V for 90 minutes using the wet electrotransfer method. Membrane blocking was performed with 3% bovine serum albumin (BSA) (Thermo Scientific) in 1X Tris-buffered saline with 0.1% Tween 20 (TBST) for 90 minutes at room temperature with gentle shaking. The membranes were then incubated overnight at 4 °C with the primary antibodies diluted in 3% BSA in TBST, which were PPAR-γ (1:500 (*v*/*v*) dilution), TLR-4 (1:200 (*v*/*v*) dilution), or IRS-1 (1:1000 (*v*/*v*) dilution) (Santa Cruz Biotechnology, Dallas, TX). The membranes were washed 5 times, for 5 minutes each, and incubated with the secondary antibody diluted in 3% BSA in TBST (1:2000 (*v*/*v*) diluted anti-mouse for PPAR-γ and TLR-4, or 1:1000 (*v*/*v*) diluted anti-rabbit for IRS-1 (Promega, Madison, WI)) for one hour at room temperature with gentle shaking. The membranes were washed 5 times, for 5 minutes each, once more, and then detection was performed using ECL detection reagent (GE Healthcare, Quebec, Canada) according to the manufacturer’s instructions. Imaging was performed using the Chemi Genius 2 Bio Imaging System (Syngene, Frederick, MD). Protein concentrations were semi-quantified using Image J software (Image Processing and Analysis in Java, National Institute of Health) by calculating relative densities of the protein bands.

### 2.10. Immunohistochemical Analysis

White adipose tissue intended for immunohistochemical analysis was stored in 10% formalin for fixation. Fixed samples were embedded in paraffin by histotechnologists in the Department of Pathobiology, University of Guelph following the formalin fixation and paraffin embedding (FFPE) protocol. Following paraffin embedding, sections of paraffin infiltrated tissue were deparaffinized in xylene and rehydrated in ethanol. The deparaffinized tissue samples were immersed in antigen retrieval buffer pre-heated to 95 °C for 10 minutes before being washed in PBS 3 times for 5 minutes each. Tissue samples were blocked with 5% BSA in PBST at 37 °C for 30 minutes, then incubated for 1 hour at room temperature with F4/80 antibody (Santa Cruz Biotechnology) diluted 1:1000 (*v*/*v*) in 5% BSA in PBST. Following this, samples were washed, as before, before incubating for 1 hour at room temperature in secondary antibody diluted 1:1000 (*v*/*v*) in 5% BSA in PBST. Samples were washed once more before staining with 3,3’-diaminobenzidine. They were then counterstained with hematoxylin, dehydrated, and mounted [33]. Images of the slides were captured using a light microscope (Olympus Life Sciences, Toronto, Canada). The total number of adipocytes and adipocytes with the crown-like structure of macrophage infiltration were counted for each slide and the percentage of adipocytes with macrophage infiltration were calculated.

### 2.11. Feces Collection

Fecal samples from each group were collected at 0, 4, and 16 weeks and then were stored at −80°C until DNA extraction.

#### 2.11.1. DNA Extraction

Bacterial DNA was extracted from fecal samples using the Qiagen QIAamp DNA stool mini kit (Qiagen, Mississauga, ON, Canada) according to the manufacturer’s “Isolation of DNA from Stool for Pathogen Detection” protocol. Briefly, 200 mg feces were mixed with buffer and InhibitEX to remove many compounds that could degrade DNA and inhibit downstream enzymatic reactions. After vortex and centrifugation at 10,000× *g* for 1 min, the DNA in the supernatant was purified on QIAamp Mini spin columns. DNA was bound to the QIAamp membrane by centrifugation and eluted from the QIAamp Mini spin column in a low-salt buffer. The overall quality and quantification of total DNA were assessed using the NanoDrop™ 8000 Spectrophotometer (Thermo fisher, Wilmington, DE, USA) and 1.5 μL of each sample was used for each measurement. Samples were stored at −20 °C until further treatment. DNA concentrations were diluted to a final concentration of 12.5 ng/μL for PCR amplification. The quality of DNA was determined by calculating A260/A280 ratio and was considered pure between 1.8 and 2.2.

#### 2.11.2. Primer Preparation

A set of 16S rRNA gene-targeted group-specific primers were designed for microbial identification, including *B. bifidum*, *L. casei*, *E. coli*, *C. difficile*, and *B. fragilis*, using GenBank (http://www.ncbi.nlm.nih.gov/) and synthesized by the University of Guelph Laboratory Services Molecular Biology Section (Guelph, Ontario, Canada) (Supplemental Table S4). Results were expressed as fold changes relative to the negative control group and relative microbial population expressions were quantified using the 2^−ΔΔCt^ method [34].

#### 2.11.3. Analysis of Fatty Acids by Gas Chromatography (GC)

Gas chromatography (GC) was used to determine fecal volatile fatty acid concentrations. Mice fecal samples were prepared and analyzed as recently described [35]. Briefly, 300 mg of freeze-dried samples were collected and freeze dried for two days. Three-milliliter MilliQ water was added and mixed by vortex to get 10% (*w*/*v*) homogenous fecal solution. Five mol/L HCl was then added to adjust pH to 2–3 and kept for 10 min at room temperature. The solution was vortexed and centrifuged at 10,000× *g* for 10 min. Next, 50 μL 2-ethylbutyric acid (Aldrich, #109959) was added to the supernatant as an internal standard. The supernatant was filtered through a 0.2 μm PVDF syringe filter and injected (1μL) into the gas chromatograph. Chromatographic analysis was carried out using Hewlett Packard 5890 Series II system equipped with J & W columns (CP-Wax 52 CB 30 × 0.53) (Agilent Technologies, Amstelveen, The Netherlands). Injector and detector temperatures were maintained at 240 °C and 280 °C, respectively. After an initial period of 1 min at 75 °C, the oven temperature was increased to 180 °C at a rate of 6 °C/min and then increased by 10 °C/min and held at 230 °C for 6 min. The final oven temperature was increased by 2 °C/min and kept at 240 °C for 5 min. The peaks were identified by comparison of their retention times with acetate, propionate, and butyrate gas chromatography standards (Sigma–Aldrich).

### 2.12. Statistical Analysis

Results are presented as mean values ± SEM (standard error of the mean). Data were analyzed using one-way analysis of variance (ANOVA) followed by one-tailed, unpaired Student’s t-tests, if two groups were compared, or Tukey’s multiple-comparison test if more than two groups were compared (GraphPad Prism version 5.0, La Jolla, CA). Differences were considered significant if the *p*-value < 0.05 (*), 0.01 (**) or 0.0001 (***).

## 3. Results

### 3.1. Basic Physiological Measures Obtained from Mice During the Trial

The average water consumption (mL mouse/day), food consumption (g mouse/day), and body weight (g) measured throughout the trial showed no significant differences between the negative control (NC), positive control (PC), or the three treatment groups (TL, TM, TH). There were no significant differences in any of the tissue weights between groups (Supplemental Table S5).

### 3.2. The Effect of IMD on Plasma Levels of Pro-Inflammatory Mediators

**Figure 1:**
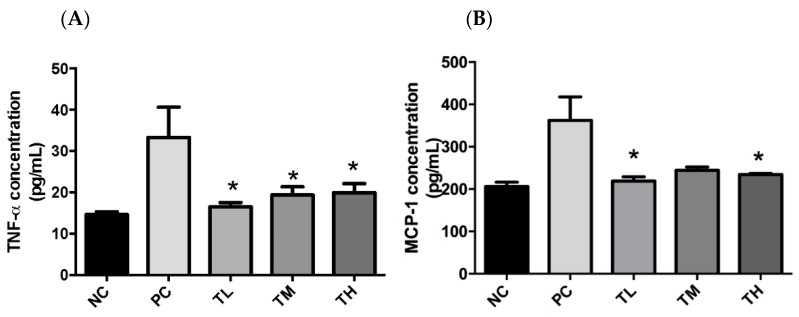
Effect of IMD on pro-inflammatory cytokine expression in the plasma of mice with LPS-induced chronic inflammation. Protein expression of TNF-α (**A**) and MCP-1 (**B**) was measured by ELISA. Results are expressed as means ± SEM of *n* = 4 samples for NC and PC, and *n* = 6 samples for TL, TM, and TH. Differences in means were considered statistically significant for *p* < 0.05. *p < 0.05 relative to PC.

When the bacterial membrane is lysed, LPS is released causing a cascade of inflammatory responses in the gut and increased gut permeability. The presence of LPS in the bloodstream increases the risk of developing endotoxemia and associated systemic inflammatory disorders [36,37]. Due to a limited quantity of plasma, samples were pooled between two mice from the same cage. Therefore, NC and PC each had four samples, TL, TM, and TH had six samples each. The plasma concentration of TNF-α for the PC group (33.37 ± 7.33 pg/mL) was significantly higher than that of the NC group (14.52 ± 0.711 pg/mL), the TM group (19.38 ± 2.00 pg/mL), or the TH group (19.94 ± 2.21 pg/mL) (Figure 1). The TNF-α plasma concentration for the TL group (16.50 ± 1.06 pg/mL) was significantly lower than that of the PC group (*p* =0.037). There was no dose-dependent effect. Plasma concentrations of MCP-1 were significantly different from the PC group (362.3 ± 55.4 pg/mL) for the NC group (206 ± 10.33 pg/mL) or the TM group (244.6 ± 7.6 pg/mL), but the TL group (235.5 ± 7.2 pg/mL, *p* = 0.037) and the TH group (234.5 ± 2.4 pg/mL, *p* = 0.036) were significantly lower relative to the PC group (Figure 1), but there is a lack of dose-dependent results. 

### 3.3. The Effect of IMD on Plasma Concentrations of Adiponectin

**Figure 2:**
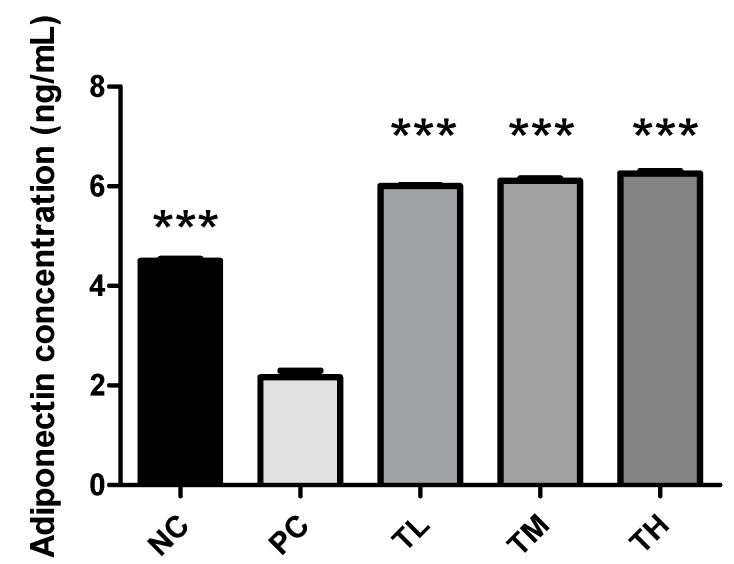
Effect of IMD on secretion of adiponectin in plasma of mice with LPS-induced chronic inflammation. Results are expressed as means ± SEM of *n* = 4 samples for NC and PC, and *n* = 6 samples for TL, TM, and TH. Differences in means were considered statistically significant for ***p < 0.001 relative to PC.

Adiponectin is one of the important adipokines and possesses the anti-inflammatory property that can activate macrophages (alternative pathway) to produce anti-inflammatory cytokines [38]. The concentration of adiponectin was significantly decreased for the PC group (2.165 ± 0.134 ng/mL) relative to the NC group (4.505 ± 0.035 ng/mL, *p* < 0.0001), TL group (6.004 ± 0.018 ng/mL, *p* < 0.001), TM group (6.109 ± 0.048 ng/mL, *p* < 0.001), and TH group (6.256 ± 0.048 ng/mL, *p* < 0.001) when compared by Tukey’s multiple means comparison (Figure 2).

### 3.4. Effect of IMD on Plasma Endotoxin Concentration

**Figure 3:**
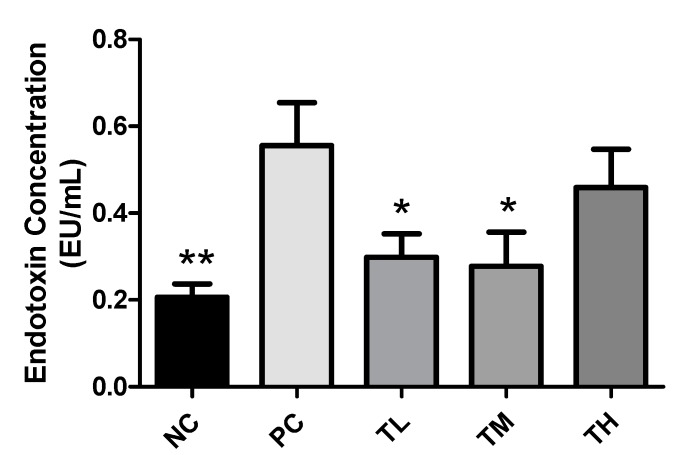
Effect of IMD on levels of endotoxin in plasma of mice with LPS-induced chronic inflammation. Results are expressed as means ± SEM of *n* = 4 samples for NC and PC, and *n* = 6 samples for TL, TM, and TH. Differences in means were considered statistically significant for *p < 0.05, **p < 0.01 relative to PC.

Endotoxin level was significantly increased for the PC group (0.6177 ± 0.0913 EU/mL) relative to the NC group (0.2063 ± 0.0309 EU/mL, *p* < 0.01). There was a significant decrease in the plasma concentrations of endotoxin for the TL group (0.2987 ± 0.0534 EU/mL, *p* < 0.05) and the TM group (0.2777 ± 0.0783 EU/mL, *p* < 0.05) relative to the PC group, but the TH group (0.4588 ± 0.0883 EU/mL) was not significantly different when compared by Tukey’s multiple means comparison (Figure 3).

### 3.5. Effect of IMD on Expression of Tight Junction Proteins and Mucin

**Figure 4:**
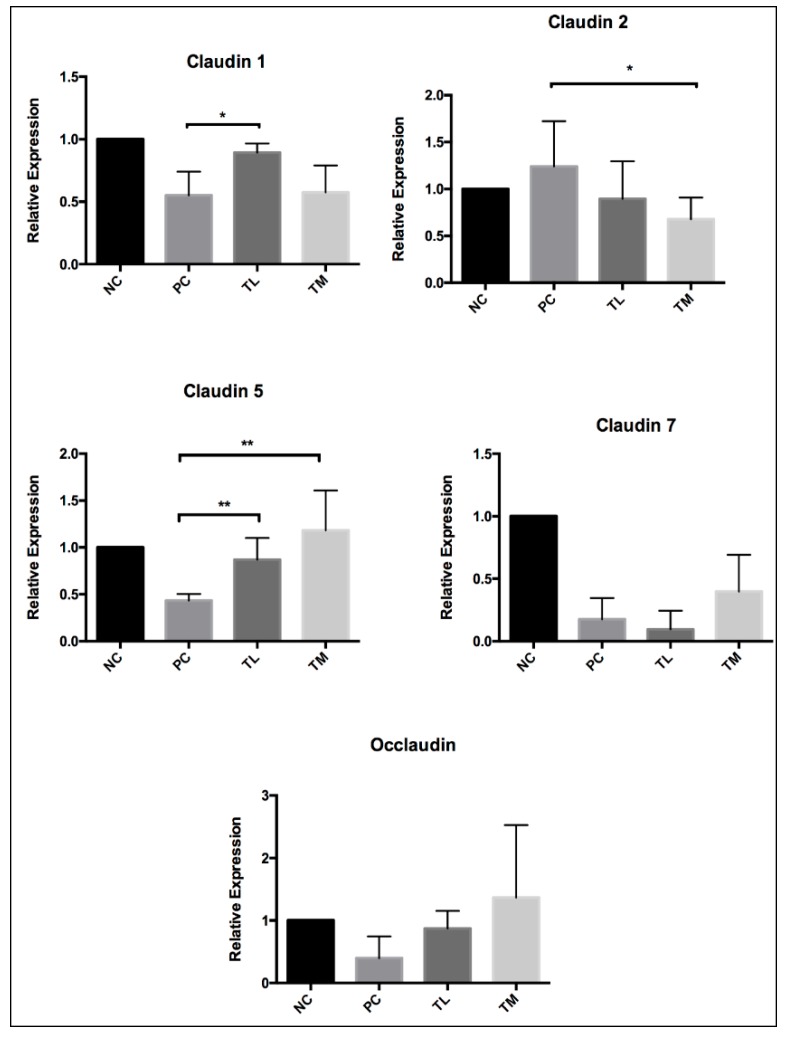

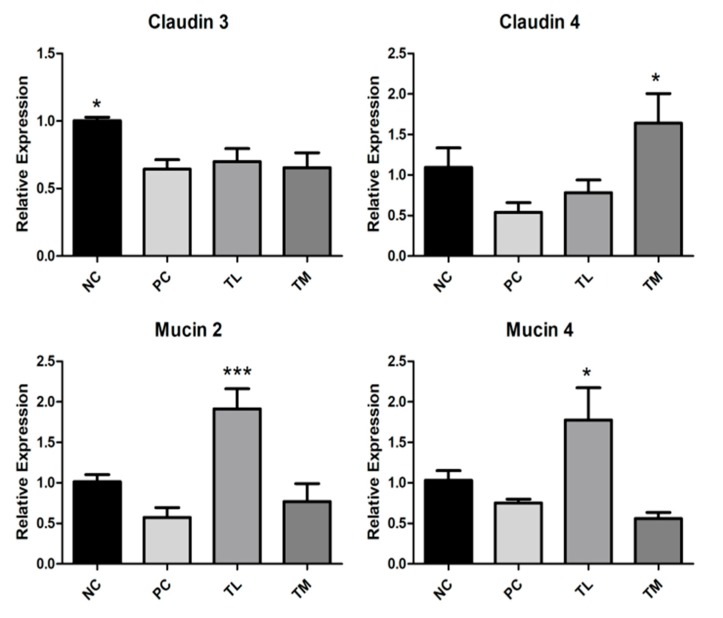
Effect of IMD on expression of tight junction proteins Claudin 1-5, 7, occlaudin, as well as mucin 2 and mucin 4 relative to GAPDH, as determined by RT-PCR. Results are expressed as mean ± SEM for *n* = 6 samples for each group. Differences between means were considered significant for *p < 0.05, **p < 0.01, or ***p < 0.001 relative to PC.

Expressions of a variety of tight junction proteins are key indicators of gut mucosal integrity. The NC group (1.003 ± 0.024) had significantly higher expression of claudin 3 than the PC group (0.6433 ± 0.0701, *p* < 0.05), although the TL group (0.7000 ± 0.0955) and the TM group (0.6550 ± 0.1090) were not significantly different from the PC group. For claudin 4, the TM group (1.642 ± 0.363) had a significantly higher expression than the PC group (0.5400 ± 0.1193, *p* < 0.05), while the NC group (1.095 ± 0.238) and TL group (0.7817 ± 0.1571) were not significantly different from the PC group. For both mucin 2 and mucin 4, the TL group (1.913 ± 0.248 and 1.777 ± 0.397, respectively) had a significantly higher expression than the PC group (0.5750 ± 0.1197, *p* < 0.001, and 0.7533 ± 0.0466. *p* < 0.05, respectively). Neither the NC group, nor the TM group were significantly different from the PC group for both mucin 2 (NC 1.015 ± 0.087; TM 0.7700 ± 0.2208) and mucin 4 (NC 1.032 ± 0.119; TM 0.5583 ± 0.0754). The claudin 1 and 5 expression of treatment groups was higher than that of the PC group, however, claudin 2 showed an opposite trend (Figure 4). 

### 3.6. Effect of IMD on PPAR-γ and IRS-1 Expression1 in the White Adipose Tissue

**Figure 5:**
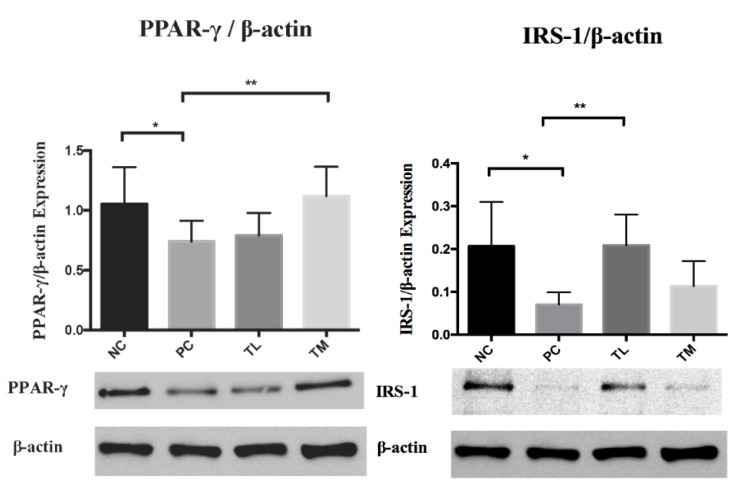
Effect of IMD on PPAR-γ and IRS-1expression in white adipose tissue of mice as determined by Western blot. Results are expressed as mean ± SEM for *n* = 4 samples per group. Differences in means were considered statistically significant for *p < 0.05 or **p < 0.01.

PPARγ plays a critical role in the integrated regulation of the immune-metabolism balance, including glucose homeostasis. TNF-α has been found to suppress PPARγ expression in mature adipocytes by upregulating MAP4K4. Furthermore, a lower expression of adaptor protein IRS-1 can interfere with the insulin signaling pathway to diminish insulin sensitivity and functional glucose uptake. [39,40]. Western blot was used to measure expression of PPAR-γ relative to β-actin in white adipose tissue of C57BL/6NCrl mice with LPS-induced chronic inflammation. There were significant differences in means of the NC, TL, or TM groups relative to the PC group (Figure 5). As shown in Figure 5, PC showed a decreased white adipose tissue IRS-1 expression compared to NC group. There was a significant increase of IRS-1 expression in IMD TL intervention group (*p* < 0.01) comparing to PC group. TM group also showed increases in IRS-1 expression comparing to PC group, but the difference was not statistically significance.

### 3.7. Macrophage Infiltration

**Figure 6:**
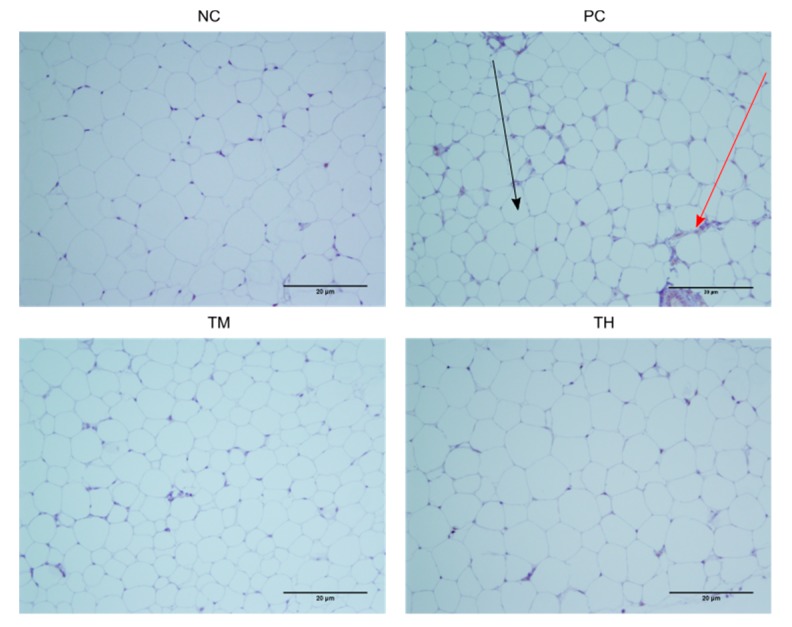
F4/80 stained immune-histochemical images of white adipose tissue. The black arrow indicates an adipocyte, while the red arrow indicates the brown-stained crown-like structure of the site of macrophage infiltration. For PC, *n* = 4 samples, and for NC, TM, and TH, *n* = 4 samples had slides made.

**Figure 7:**
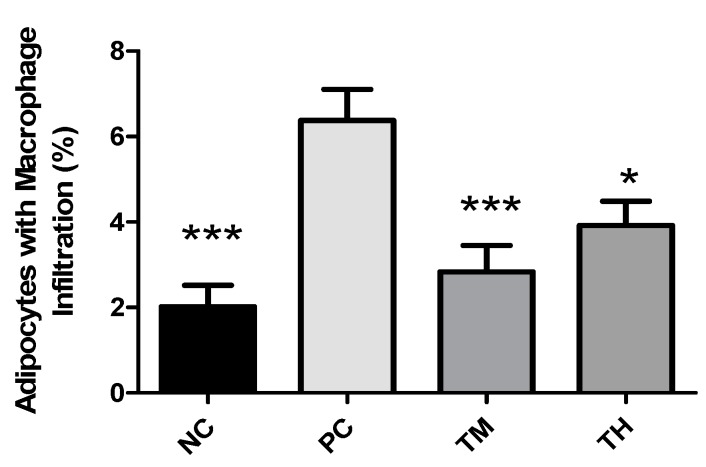
Percentage of adipocytes with macrophage infiltration in adipose tissue of mice with LPS-induced chronic inflammation. Results are expressed as means ± SEM. Differences in means were considered statistically significant for *p < 0.05, or ***p < 0.001 relative to PC.

A dysfunctional WAT typically shows hypertrophy of adipocytes accompanied by macrophage infiltration, resulting in an increased production of proinflammatory mediators. Macrophage infiltration appears as a brown crown-like structure surrounding adipocytes. The percentage of adipocytes with macrophage infiltration was determined (Figure 6 and Figure 7). There was a significant increase in the percent infiltration in the PC group (6.375 ± 0.728%) relative to the NC group (2.016 ± 0.499%, *p* < 0.001). Relative to the PC group, the percent infiltration was decreased in the TM group (2.832 ± 0.620%, *p* < 0.001) and the TH group (3.914 ± 0.572%, *p* < 0.05). 

### 3.8. TLR4 Expression

Western blot was used to measure the expression of TLR4 relative to β-actin in white adipose tissue of mice with LPS-induced chronic inflammation. There were no significant differences between means of the NC group (1.140 ± 0.240), PC group (1.063 ± 0.145), TL group (1.226 ± 0.117), or TM group (1.108 ± 0.184) (Supplemental Figure S2).

### 3.9. Effect of IMD on Glucose Tolerance and Insulin Resistance

#### 3.9.1. Glucose Tolerance Test

**Figure 8:**
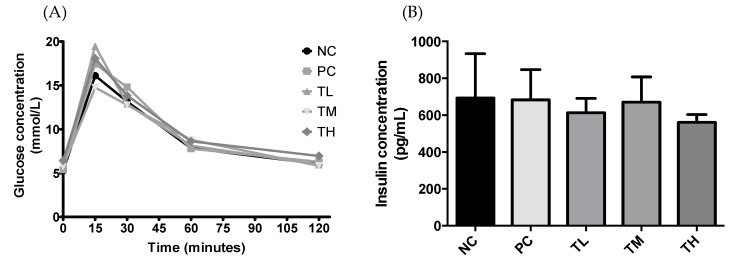
Effect of IMD on blood glucose levels (**A**) and insulin concentration (**B**) in the plasma of mice with LPS-induced chronic inflammation, as measured by ELISA. Results are expressed as means ± SEM.

At the baseline, glucose concentrations were very similar between groups, at approximately 5 mmol/L. The glucose concentrations showed more variability when measured at 15 minutes, with the TM group (14.750 ± 0.959 mmol/L) having the lowest glucose level, followed by NC (16.108 ± 1.190 mmol/L). The TL treatment group (19.467 ± 0.958 mmol/L) had the highest blood glucose concentration, closely followed by the TH group (18.067 ± 1.560 mmol/L) and the PC group (17.433 ± 0.509 mmol/L). The blood glucose concentrations for most of the treatment groups was around 13 mmol/L at 30 minutes following the gavage, although the PC treatment group had the highest glucose level at 14.800 ± 1.049 mmol/L (Figure 8). However, there were no statistically significant differences between any of the treatment groups as determined by Tukey’s multiple means comparison.

#### 3.9.2. Plasma Insulin Concentration

Plasma insulin concentration was measured for the mice that underwent the glucose tolerance test. Plasma from the two mice of each cage was pooled, so the plasma sample size was 2 for NC and PC and 3 for TL, TM, and TH. There were no significant differences between the NC (693.0 ± 241.0 pg/mL), PC (683.0 ± 164.0 pg/mL), TL (612.7 ± 78.52 pg/mL), TM (670.0 ± 136.9 pg/mL), or TH (561.0 ± 42.44 pg/mL) groups (Figure 8).

#### 3.9.3. Effect of IMD on Intestinal Permeability

**Figure 9:**
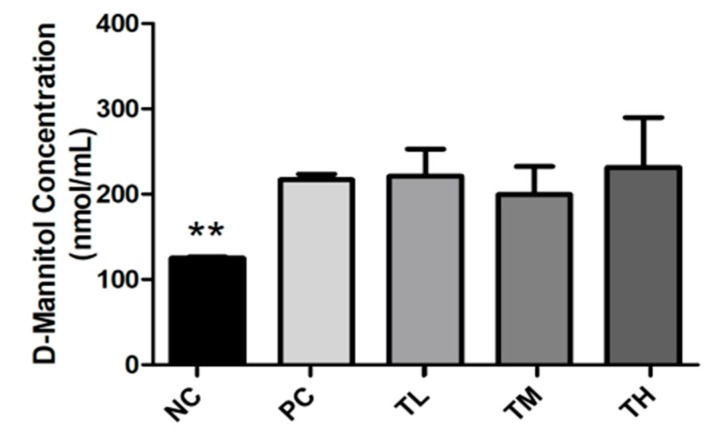
Effect of IMD on D-mannitol concentration in the plasma of mice with LPS-induced chronic inflammation, as measured by colorimetric assay. Results are expressed as means ± SEM of *n* = 2 samples for NC and PC, and *n* = 3 samples for TL, TM, and TH. Differences in means were considered statistically significant for **p < 0.01 relative to PC.

D-mannitol was used as an indicator of leaky gut to assess the integrity of the intestinal barrier. Plasma samples for the two or three mice from each cage that underwent the D-mannitol gavage were used to determine D-mannitol concentrations. The NC group (125.00 ± 1.40 nmol/mL) had a significantly lower D-mannitol concentration than the PC group (216.80 ± 6.66 nmol/mL, *p* < 0.01) (Figure 9). The treatment groups TL (221.0 ± 31.9 nmol/mL), TM (199.4 ± 33.2 nmol/mL), and TH (231.4 ± 58.32 nmol/mL) were not significantly different from the PC group.

#### 3.9.4. Effect of Prebiotic IMD on Relative Microbiota Population

**Figure 10:**
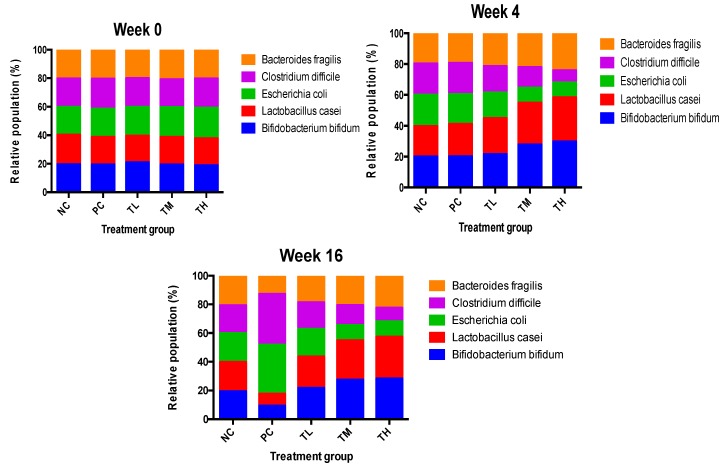
Effect of prebiotic IMD on the relative percentage change of each bacterium compared to the total bacteria population in LPS-induced low-grade inflammatory mice. NC, negative control (untreated mice); PC, positive control (mice treated with LPS); TL, treatment low (mice treated with 1% IMD and LPS); TM, treatment medium (mice treated with 2.5% IMD and LPS); TH, treatment high (mice treated with 5% IMD and LPS). The stacked bar graph is presented as the relative percentage of *B. bifidum, L. casei, E. coli, C. difficile,* and *B. fragilis* on week 0, 4, and 16 (*n* = 6).

The microbiota plays a major role in many metabolic functions, including modulation of glucose and lipid homeostasis, regulation of satiety, production of energy and vitamins, and intestinal immune system [41,42]. The effect of the prebiotic IMD on the relative population of five bacterial species was analyzed by qRT-PCR. The results were expressed as relative composition analysis. As shown in Figure 10, the prebiotic IMD had a strong impact on gut microbiota that the percentage of *B. bifidum*, *L. casei,* and *B. fragilis* in IMD treatment groups increased significantly, while *E. coli* and *C. difficile* was reduced from week 0 to week 4 (Figure 10). Specifically, the relative population of *B. bifidum* and *L. case* increased from 19% to 28% and 19% to 27%, respectively, in the TM group, but *E. coli* and *C. difficile* decreased from 21% to 10% and 21% to 13% respectively. The ability of IMD to alter intestinal microflora varied, depending on different doses of IMD. The percentage of beneficial bacteria in the TM group was 56%, which was similar to that in the TH group. Thus, the TM group revealed the most optimal effect when compared to the TL and TH groups. As shown in Figure 10, LPS inhibited the growth of *B. bifidum*, *L. casei,* and *B. fragilis*, but promoted the growth of *E. coli* and *C. difficile* in the PC group from week 4 to week 16. In the PC group, the proportion of *B. bifidum* and *L. casei* decreased from 21% to 9% and 20% to 9%, respectively, while the percentage of *E. coli* and *C. difficile* increased from 20% to 34% and 20% to 35%, respectively. Compared to the PC group, the IMD treatment groups maintained the predominant level of *B. bifidum*, *L. casei,* and *B*. *fragilis* with a small percentage of *E. coli* and *C. difficile* (Figure 10). Twelve weeks of LPS treatment did not reduce the beneficial bacteria. To some extent, the prebiotic IMD could alleviate the damage of LPS to the gut intestine and optimize the intestinal microflora composition.

#### 3.9.5. The Levels of SCFAs in Mice Feces Analysis

It was reported that IMD can be fermented in the colon in rat [30], however, its profile of fermented metabolites has not been studied. In this study, we measured SCFAs. Table 2 shows the concentrations of acetic acid, propionic acid, and butyric acid in the NC, the PC, the MD treated medium group at weeks 0, 4, and 16. Butyric acid had the highest concentration, followed by acetic acid and propionic acid. As shown in Table 2, compared to the NC group (3.73 ± 0.74 μg/mL and 12.13 ± 0.04 μg/mL), there was a significant decrease in the concentration of acetic acid (1.63 ± 0.17 μg/mL) and butyric acid (5.33 ± 1.32 μg/mL) in the PC group. The IMD treated medium group showed a significant increase of acetic acid (4.39 ± 1.55 μg/mL) and butyric acid (15.10 ± 1.39 μg/mL), compared to the PC group at week 16. The IMD treated medium group showed no significant difference in the concentration of propionic acid. 

## 4. Discussion

There is growing awareness that prolonged low-grade chronic inflammation is a primary causative factor for the increased risk of developing chronic conditions, including metabolic disorders and cancers. The first objective of this study was to determine if IMD could exert an anti-inflammatory effect in C57BL/6NCrl mice with LPS-induced low-grade chronic inflammation, as well as to elucidate the potential mechanism of action and evaluate the effectiveness of IMD at preventing inflammation-induced metabolic disorders. As an inflammatory inducer, LPS is known to increase production of pro-inflammatory mediators (e.g. TNF-α, IL-6, and MCP-1) and to inhibit the activity of the anti-inflammatory mediator adiponectin [43]. In fact, the plasmatic pro-inflammatory cytokines, TNF-α and MCP-1, were significantly increased by LPS in the PC group (Figure 1). Furthermore, 1% IMD had significantly lowered plasma concentrations of TNF-α relative to the group with administration of LPS alone (Figure 1). This suppression of TNF-α levels agrees with a previous study during, in which the effect of IMD on a DSS-induced mouse model of colitis was investigated [31]. TNF-α is a potent pro-inflammatory cytokine, as it can activate the NF-κB pathway, which induces the production of additional pro-inflammatory cytokines [7,44]. The mice treated with 1% IMD and 5% IMD in addition to LPS had significantly lower levels of MCP-1 relative to mice treated with LPS alone (Figure 1). MCP-1 is a chemokine that induces the migration of monocytes and lymphocytes to areas of inflammation and is another pro-inflammatory mediator [45]. 

Adiponectin is produced by adipocytes and can exert an anti-inflammatory effect [13]. High levels of adiponectin are associated with an decreased expression of pro-inflammatory mediators, such as TNF-α, IL-6, and MCP-1, and an increased expression of the anti-inflammatory cytokine IL-10 [13]. Adiponectin can also inhibit the NF-κB pathway and enhance the expression of PPAR-γ2, which can suppress the trans-activational ability of NF-κB [13]. In the current study, plasma concentrations of adiponectin were significantly increased in mice that received 1%, 2.5%, and 5% IMD in addition to LPS relative to mice that received LPS alone (Figure 2). The decrease in plasma concentrations of pro-inflammatory mediators TNF-α and MCP-1, along with the increase of the anti-inflammatory mediator adiponectin in mice administered IMD, strongly suggests that this treatment may be capable of exerting an anti-inflammatory effect on C57BL/6NCrl mice with LPS-induced low-grade chronic inflammation. Endotoxin, or LPS, can interact with TLR4 to directly activate the NF-κB pathway and increase the expression of pro-inflammatory mediators, such as TNF-α, IL-6, and MCP-1 [45]. Increased circulating levels of endotoxin, potentially due to increased bacterial endotoxin translocation caused by a loss of intestinal barrier integrity or exogenous administration of LPS, could therefore promote inflammation. This decrease in plasma endotoxin levels in IMD treatment may be one of the anti-inflammatory mechanisms of action of IMD. The expression of PPAR-γ is enhanced by adiponectin and suppressed by LPS via activation of TNF-α [13,46]. There were no statistically significant differences between the treatment groups, although the groups administered IMD and LPS showed a slight tendency toward increased expression of PPAR-γ (Figure 5). The lack of significance may be due to a large standard error, although it may also indicate that the anti-inflammatory mechanism of action of IMD may lie elsewhere. Previous studies have also found that resistant starches can modulate the expression of toll-like receptors, and in this way, lower the expression of pro-inflammatory cytokines [47,48]. A previous study by Majumder et al., in a DSS-induced mouse model of colitis treated with IMD, found evidence that treatment with IMD in conjunction with DSS could decrease the expression of TLR4 compared to treatment with DSS alone [31]. The expression of TLR4 in the white adipose tissue was examined by Western blot in the present study. In the present study, the group receiving LPS alone did not demonstrate any increase in the expression of TLR4 relative to the group receiving water, which suggests that the current model of low-grade inflammation may have been insufficient to induce an increase in TLR4 expression. Therefore, in the current trial, TLR4 expression is not a likely mechanism for IMD to exert an anti-inflammatory effect.

Macrophage infiltration is promoted by a high concentration of MCP-1 in white adipose tissue, which recruits monocytes from circulation to the adipose tissue, where they differentiate into M1 macrophages [11,49,50]. Macrophages in white adipose tissue are responsible for much of the pro-inflammatory cytokines produced by that tissue, including TNF-α and IL-6, hence, increased infiltration of macrophages into white adipose tissue would also promote inflammation [11]. In the present study, mice from groups treated with 2.5% and 5% IMD in conjunction with LPS had a significantly lower percentage of adipocytes with macrophage infiltration compared to the group receiving LPS alone (Figure 7). This provides a potential mechanism for the reduction of pro-inflammatory cytokines and anti-inflammatory effect of IMD because fewer macrophages infiltrating the white adipose tissue would reduce the secretion of pro-inflammatory cytokines, such as TNF-α.

The mucus layer in the intestines is a first line of defense against pathogens in the gastrointestinal tract. A lack of functioning mucin 2 has been demonstrated to contribute to inflammation [51]. The gene expression of mucin 2 and mucin 4 was significantly higher in mice treated with 1% IMD with LPS compared to mice receiving LPS alone (Figure 4). This indicates that IMD may act in an anti-inflammatory manner, by improving the mucosal integrity. The TJ complex is essential for maintaining the selective permeability and integrity of the paracellular pathway. Claudin 1 and 5 were restored by the IMD intervention and claudin 2 was suppressed at 2.5% IMD treatment. Conversely, expression of claudin 4 was significantly increased in the group of mice receiving 2.5% IMD and LPS relative to the mice receiving LPS alone (Figure 4). Claudin 4 promotes the tightening of TJ complexes and decreases paracellular permeability [52]. Treatment with IMD could potentially improve intestinal permeability by increasing expression of claudin1, 4, and 5, while suppressing claudin 2. 

Adiponectin can act as an insulin sensitizer, while pro-inflammatory cytokines, such as TNF-α and IL-6, contribute to insulin resistance [10,11]. Since treatment with IMD was demonstrated in this study to promote the expression of adiponectin and reduce the expression of TNF-α, it follows that IMD may also have an impact on insulin resistance. There were no significant differences in either plasma insulin concentration or in glucose (Figure 8) between any of the treatment groups, including between the group receiving just water and the group receiving LPS only. This indicates that the low-grade inflammation induced by LPS may have been insufficient to induce insulin resistance in a mouse model, making it difficult to evaluate the effectiveness of IMD on preventing insulin resistance. A previous clinical trial by Sadakiyo et al. in 30 healthy adult men and women found that the postprandial change in blood glucose levels was significantly decreased at 45 minutes after glucose loading, when 5 g of IMD (2.5%) was added to glucose load, when compared to the substances on their own, but the difference did not reach significance when 10 g (5%) of IMD was administered [29]. Administration of 9.6 g (4.8%) of IMD in a maltodextrin loading study also resulted in a significantly decreased blood glucose level at 45 minutes relative to maltodextrin administration alone [29]. This suggests that IMD may have some impact on the glycemic response, although it was not demonstrated in the current study, hence, further research is required to verify this effect. 

There was a significant increase in the relative population of *Bifidobacterium bifidum*, *Bacteroides fragilis*, and *Lactobacillus casei* and a significant decrease in the relative population of *Escherichia coli* and *Clostridium difficile* in all groups treated with IMD and LPS compared to groups receiving LPS alone in week 16 but not in week 0. *C. difficile* and *E. coli* are typically harmful to the host, while *Lactobacillus* and *Bifidobacterium* generally considered to be beneficial and are frequent targets of both probiotic and prebiotic treatment [53]. *Bifidobacterium* have also been shown to improve intestinal barrier function [54,55]. Furthermore, acetic acid and butyric acid levels were significantly increased in week 16 for mice receiving 2.5% IMD and LPS compared to mice receiving LPS alone. Butyric acid is an important energy source for colonocytes, inhibits the expression of pro-inflammatory mediators, and may inhibit the NF-κB pathway [56,57]. It has also been demonstrated that treatment with acetate reduced production of TNF-α by LPS-stimulated neutrophils and IL-6 production by colon organ cultures with DSS-induced inflammation [58]. Acetate and butyrate have also been shown to dose dependently reduce the activity of NF-κB in TNF-α-stimulated cells [59]. Therefore, treatment with IMD could exert an anti-inflammatory effect in this manner.

Further studies on bioaccessibility and bioavailability of IMD should be considered in connection to anti-inflammatory activity of IMD and prevention of metabolic disorders. IMD consists of low molecular alpha-glucan (average molecular weight is about 3500) which is resistant to GI digestive system. Although it is partially digested into glucose, mostly, it is considered to remain in the colon, which is a typical prebiotic characterization. It is considered that IMD is not absorbed from the intestines, but could modulate microbiota and exhibits an anti-inflammatory activity in the gut. 

## 5. Conclusions

Chronic exposure to a low-dose of endotoxin triggers pronounced inflammatory responses impairing gut physical barrier and resulting in microbiota dysbiosis. In this study, we investigated the anti-inflammatory activity of IMD in a LPS-induced low-grade chronic inflammation C57BL/6NCrl mouse model. Administration of IMD exhibited an anti-inflammatory effect by lowering plasma concentrations of the pro-inflammatory mediators TNF-α and MCP-1 and increasing concentrations of adiponectin, an anti-inflammatory adipokine with a reduction in macrophage infiltration in WAT. The results also show that the deficient expression of tight junction protein (Cldn 4) and mucin 2 and 4 were restored by IMD as well as decrease in plasma concentrations of endotoxin. The dysbiosis in gut microbiota were prevented by IMD. These findings suggest that IMD exerts protective effects on intestinal mucosal integrity and on an early risk for progression into metabolic disorders. 

## Figures and Tables

**Table 1:** Experimental design for the animal trial.

	Pre-treatment				Intervention											
Week	1	2	3	4	5	6	7	8	9	10	11	12	13	14	15	16
NC	Autoclaved water															
PC	Autoclaved water				LPS in autoclaved water											
TL	1% IMD in autoclaved water				LPS and 1% IMD in autoclaved water											
TM	2.5% IMD in autoclaved water				LPS and 2.5% IMD in autoclaved water											
TH	5% IMD in autoclaved water				LPS and 5% IMD in autoclaved water											

Note: Treatment groups are referred to as follows: NC (negative control), PC (positive control), TL (low treatment dose), TM (medium treatment dose), and TH (high treatment dose). All mice received Teklad Global 14% mouse maintenance diet from Envigo for the duration of the trial. Throughout the trial, mice in the NC and PC groups received autoclaved water, while mice in the TL, TM, and TH groups received 1%, 2.5%, and 5% isomaltodextrin (IMD) in autoclaved water, respectively. Following the pre-treatment period, LPS was added to the drinking water at the beginning of week 5 in a dosage of 300 μg lipopolysaccharide (LPS) (kg body weight)/day to mice in the PC, TL, TM, and TH groups to induce chronic low-grade inflammation.

**Table 2:** Concentrations of acetic, propionic, and butyric acids in fecal samples from the IMD-treated mouse trial.

SCFA/Concentration (μg/mL)		Acetic Acid	Propionic Acid	Butyric Acid
**Week 0**	NC	2.81 ± 0.79 a	0.49 ± 0.18 a	14.63 ± 2.47 a
	PC	3.17 ± 0.37 a	0.73 ± 0.03 a	13.57 ± 0.15 a
	TM	2.57 ± 0.71 a	0.62 ± 0.18 a	13.87 ± 2.80 a
**Week 4**	NC	3.92 ± 0.61 a	0.71 ± 0.02 a	11.80 ± 0.44 a
	PC	2.39 ± 0.45 a	0.31 ± 0.06 a	12.31 ± 1.31 a
	TM	5.56 ± 0.60 b*	0.81 ± 0.10 a	16.77 ± 2.80 a
**Week 16**	NC	3.73 ± 0.74 a*	0.55 ± 0.06 a	12.13 ± 0.04 a*
	PC	1.63 ± 0.17 b	0.74 ± 0.06 a	5.33 ± 1.32 b
	TM	4.39 ± 1.55 b*	0.59 ± 0.13 a	15.10 ± 1.39 a*

Values are expressed in mean ± standard deviation (SD) of acetic acid, propionic acid, and butyric acid. NC, negative control (untreated mice); PC, positive control (mice treated with LPS); TM, treatment medium (mice treated with 2.5 % IMD and LPS). The significant differences are shown by asterisk, *p < 0.05, compared with the positive control group. The different letters showed the significant differences under treatment (*p* < 0.05).

## Supplementary Materials

The following are available online at https://www.mdpi.com/2072-6643/11/11/2791/s1, Table S1: cDNA synthesis thermal cycling conditions. Table S2: RT-PCR thermal cycling conditions. Table S3: Primer sequences used in this study for tight junction analysis. Table S4: Primers used in PCR analysis of microbiota analysis. Table S5: Changes of body weight and tissues weight collected at euthanasia. Data was presented as mean ± SEM. Figure S1: Putative structure of isomaltodextrin, an α-glucan resistant to digestion. (a) Nonreducing end α-D-Glc; (b) 1,6-linked α-D-Glc; (c) 1,3-linked α-D-Glc; (d) 1,3,6-linked α-D-Glc; (e) 1,4-linked α-D-Glc; (f) 1,4,6-linked α-D-Glc; (g) reducing end α-D-Glc. Figure S2: Effect of IMD on TLR4 expression in white adipose tissue of mice with LPS-induced chronic inflammation, as determined by western blot. Results are expressed as mean ± SEM for *n* = 6 samples per group. Differences in means were considered statistically significant for *p* < 0.05.

## Abbreviations

IMD	Isomaltodextrin
NC	Negative control
PC	Positive control
TL	low treatment dose
TM	medium treatment dose
TH	high treatment dose
GI	gastrointestinal
TJ	tight junction
ZO-1	zonula occludens-1
LPS	lipopolysaccharide
PAMP	pathogen-associated molecular pattern
M cell	microfold cell
SCFA	short chain fatty acid
NF-κB	nuclear factor kappa-light-chain enhancer of activated B cells
IL	interleukin
TNF	tumor necrosis factor
MCP-1	monocyte chemoattractant protein-1
NAFLD	non-alcoholic fatty liver disease
WAT	white adipose tissue
PPAR-γ	peroxisome proliferator activated receptor gamma
IRS	insulin receptor substrate
GLUT4	glucose transporter type 4
PUFA	polyunsaturated fatty acid
GOS	galactooligosaccharide
IMD	isomaltodextrin
ELISA	enzyme-linked immunosorbent assay
RT-PCR	real-time polymerase chain reaction
PBST	phosphate buffered saline with 0.05% Tween20
TMB	tetramethylbenzidine
LAL	*Limulus* amebocyte lysate
GAPDH	glyceraldehyde-3 phosphate dehydrogenase
SDS-PAGE	sodium dodecyl sulfate-polyacrylamide gel electrophoresis
BSA	bovine serum albumin
TBST	Tris-buffered saline with 0.1% Tween20
