# Supplementary material for: Anti-Inflammatory Activity of Isomaltodextrin in a C57BL/6NCrl Mouse Model with Lipopolysaccharide-Induced Low-Grade Chronic Inflammation
Source: Nutrients. 2019 Nov 15;11(11):2791. doi: 10.3390/nu11112791 (PMC6893451; doi:10.3390/nu11112791)
Supplement: Supplementary file 1 [file nutrients-11-02791-s001.pdf]

Supplemental Table S1. cDNA synthesis thermal cycling conditions

Settings	Temperature (°C)	Time (min)
Step 1	25	10
Step 2	37	120
Step 3	85	5
Step 4	4	◎

Supplemental Table S2. RT-PCR thermal cycling conditions

Step	Temperature (°C)	Time (s)
Denaturation	95	15
Annealing	56	15
Extension	72	30

Supplemental Table S3. Primer sequences used in this study for tight junction analysis.

Mouse gene	Forward primer sequence (5'-3')	Reverse Primer Sequence (5'-3')
GAPDH	AACTTTGGCATTGTGGAAGG	GGATGCAGGGATGATGTTCT
Claudin 2	GGCTGTTAGGCACATCCAT	TGGCACCAACATAGGAACTC
Claudin 3	AAGCCGAATGGACAAAGAA	CTGGCAAGTAGCTGCAGTG
Claudin 4	CGCTACTCTTGCCATTACG	ACTCAGCACACCATGACTTG
Occludin	AAGTCAACACCTCTGGTGCC	TCATAGTGGTCAGGGTCCGT
JAM-A	ACCCTCCCTCCTTTCCTTAC	CTAGGACTCTTGCCCAATCC
ZO-1	AGGACACCAAAGCATGTGAG	GGCATTCCTGCTGGTTACA
Mucin 2	CGACACCAGGGATTTCGCTTAAT	CACTTCCACCCTCCCGGCAAAC
Mucin 4	CTCCAAGAAATGTAGTGGCTTTCAG	CACGGTCTTGGGCTGGAGTA

Supplemental Table S4. Primers used in PCR analysis of microbiota analysis.

Bacteria	Forward primer (5'-3')	Reserve primer (5'-3')	Product (bp)	Accession No.
B. bifidum	cgtcgccttcttcttcgtct	tctcaaagagctcgtaggcg	137	EF417563
L. casei	tcgtgtcgtgagatgttggg	accttcctccggtttgtcac	113	EU715321
E. coli	gtcacggcaacaaatgctgt	atggccacaacaacgaaagc	173	D78167
C. difficile	gacccgatgatagcccttcc	ggtgcgatagtccctgttcc	146	HF930131
B. fragilis	gtagagtcatcccttcgcgg	agaactcgacaaaccgggac	104	CR626927
Eubacteria	actcctacgggaggcagcag	attaccgcggctgctgg	200	Walter, J., et al., 2006

Supplemental Table S5. Changes of body weight and tissues weight collected at euthanasia. Data was presented as mean ± SEM.

	Week						
	1 2 3	4 5 6	7 8	9 10	11 12	13 14	15
**Body Weight (g)**							
**NC**	21.05 ± 0.51	22.89 ± 0.53	23.26 ± 0.49	23.75 ± 0.53	23.92 ± 0.77	24.60 ± 0.74	25.10 ± 0.72
**PC**	20.85 ± 0.30	22.31 ± 0.31	22.51 ± 0.28	23.49 ± 0.30	22.59 ± 0.29	23.55 ± 0.57	24.48 ± 0.42
**TL**	21.19 ± 0.27	22.42 ± 0.24	23.04 ± 0.22	23.54 ± 0.24	23.58 ± 0.29	24.45 ± 0.29	24.66 ± 0.28
**TM**	21.28 ± 0.30	22.92 ± 0.30	23.68 ± 0.43	24.50 ± 0.40	23.92 ± 0.49	25.45 ± 0.46	25.45 ± 0.37
**TH**	21.69 ± 0.39	23.15 ± 0.40	23.55 ± 0.46	24.20 ± 0.45	24.16 ± 0.50	25.53 ± 0.55	25.55 ± 0.60

	**White Adipose Tissue (g)**	**Brown Adipose Tissue (g)**	**Kidney (g)**	**Liver (g)**	**Leg Muscle (g)**	**Heart (g)**
**NC**	0.920 ± 0.065	0.059 ± 0.004	0.291 ± 0.007	1.137 ± 0.034	1.695 ± 0.049	0.132 ± 0.003
**PC**	0.999 ± 0.108	0.065 ± 0.003	0.273 ± 0.005	1.086 ± 0.027	1.719 ± 0.029	0.130 ± 0.003
**TL**	0.862 ± 0.111	0.066 ± 0.005	0.301 ± 0.009	1.106 ± 0.035	1.763 ± 0.083	0.133 ± 0.002
**TM**	1.045 ± 0.084	0.062 ± 0.004	0.294 ± 0.005	1.147 ± 0.031	1.811 ± 0.069	0.124 ± 0.004
**TH**	1.154 ± 0.155	0.065 ±0.005	0.292 ± 0.006	1.171 ± 0.035	1.776 ± 0.030	

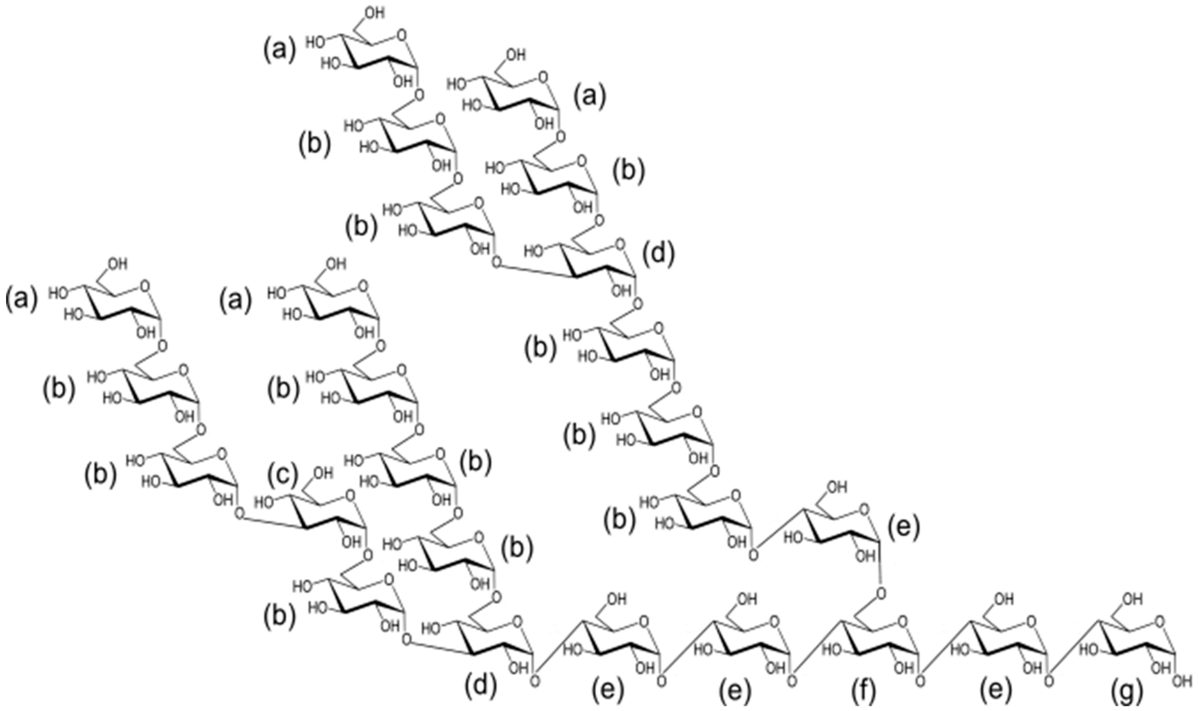

Supplemental Figure S1. Putative structure of isomaltodextrin, an α-glucan resistant to digestion. (a) Nonreducing end α-D-Glc; (b) 1,6-linked α-D-Glc; (c) 1,3-linked α-D-Glc; (d) 1,3,6-linked α-D-Glc; (e) 1,4-linked α-D-Glc; (f) 1,4,6-linked α-D-Glc; (g) reducing end α-D-Glc.

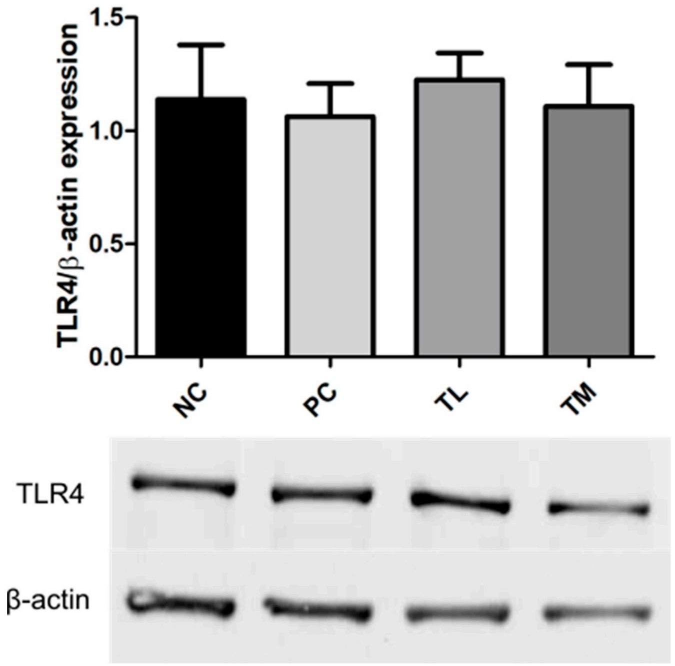

Supplemental Figure S2. Effect of IMD on TLR4 expression in white adipose tissue of mice with LPS-induced chronic inflammation, as determined by western blot. Results are expressed as mean ± SEM for n = 6 samples per group. Differences in means were considered statistically significant for $p<0.05$.
